# Effect of low-molecular-weight heparins on anti-Xa peak levels and adverse reactions in Chinese patients with recurrent spontaneous abortion: a single-center, observational study

**DOI:** 10.1186/s12884-021-04161-1

**Published:** 2021-10-07

**Authors:** Wenxin Bai, Xinyang Zhang, Si Sun, Qiaohong Wang, Congcong Li, Xiaoxin Zhang, Aimin Zhao

**Affiliations:** Department of Obstetrics and Gynecology, Ren Ji Hospital, Shanghai Jiao Tong University School of Medicine, 160 Pujian Road, Pudong District 200127 Shanghai, China

**Keywords:** Low molecular weight heparin, Recurrent spontaneous abortion, Peak anti-Xa, levels; adverse reaction.

## Abstract

**Objective:**

To compare three commonly used low-molecular-weight heparins (LWMHs) in the treatment of recurrent spontaneous abortion (RSA) by evaluating the anti-Xa peak levels and adverse reactions.

**Methods:**

In this single-center, observational study, we enrolled 310 patients with RSA in whom anti-Xa levels were measured during pregnancy. Patients were divided into three groups according to the LMWH they used: the nadroparin group, enoxaparin group and dalteparin group. We compared the peak anti-Xa levels and the coagulation status of each group, and analyzed the incidence of adverse reactions, including local allergy, liver and renal dysfunction, and the impact on platelet.

**Results:**

Patients in the enoxaparin group had a higher anti-Xa peak level than those in the nadroparin group (0.80 ± 0.22 IU/ml vs. 0.61 ± 0.24 IU/ml; *P* <  0.0001), although most patients in the three groups reached the target concentration of anti-Xa. Furthermore, patients in the enoxaparin group had a more stable anti-Xa levels during pregnancy. In addition, patients in the nadroparin group had a higher rate of local allergy than those in the enoxaparin group (60.5% vs. 42.5%; *P* = 0.004) and those in the dalteparin group (60.5% vs. 33.3%; *P* = 0.002). Further examination by the type of local allergy indicated a dramatic difference in pruritus and induration between the nadroparin group and the other two groups. No difference was found in the incidence of liver and renal dysfunction and thrombocytopenia.

**Conclusion:**

Compared with nadroparin and daltepatin, enoxaparin showed a better performance regarding anti-Xa levels and the incidence of adverse reactions in the treatment of RSA.

## Introduction

Recurrent spontaneous abortion (RSA), or recurrent miscarriage (RM), defined as two or more consecutive spontaneous abortions before the 24th week [1], is a common disease during gestation. The incidence rate is usually between 1% ~ 5% [2], and the probability of miscarriage in RSA patients can be as high as 70% ~ 80% [3]. Various factors may lead to RSA, including anatomy abnormality, chromosome abnormality, infection, prethrombotic state (PTS), autoimmune disorders and endocrine disturbance [4]. In addition, there is a large proportion of RSAs, called unexplained recurrent spontaneous abortion (URSA) [5], that cannot be explained by the above factors.

Various treatments are available for RSA, among which heparin has been recognized as an effective treatment for RSA caused by PTS and rheumatic disorders (e.g., antiphospholipid syndrome and systemic lupus erythematosus) [6]. Previous studies have shown that low-molecular-weight heparin (LMWH) not only has anticoagulant effect, but also serves as an immune regulator in promoting trophoblast invasion, inhibiting cell apoptosis, protecting vascular endothelium and promoting placental formation [7, 8]. Recently, some studies also reported that LMWHs could be used to improve the pregnancy outcome of URSA [9, 10].

Heparin can be classified as unfractionated heparin (UFH) and LMWH. LMWHs are glycosaminoglycan that comprises of 12–18 saccharide units produced by UFH through chemical methods or enzymatic depolymerization [11]. Similar to UFH, LWMHs exert their anticoagulant activity by binding to antithrombin (AT), thereby causing a conformational change at the reactive center. This transforms AT into a more efficient inhibitor of serine proteinase which can accelerate the interaction of AT with factor Xa [12]. Compared with UFH, LMWHs have a higher biological activity, longer half-life and a higher ratio of anticoagulant factor Xa/IIa, and can reduce the risk of haemorrhage [13]. Moreover, the nonspecific binding between LMWHs and macrophages, endothelial cells, osteoblasts, plasma proteins, platelets and platelet factor 4 (PF4) can reduce the incidence of heparin-related adverse reactions, such as heparin induced thrombocytopenia (HIT), osteoporosis, and heparin-induced bleeding [14]. For these reasons, LMWHs have gradually replaced UFH as the preferred therapy for the treatment of RSA.

At present, the most commonly used LMWHs include nadroparin, enoxaparin and dalteparin. Although they have similar therapeutic effects and functioning mechanisms, these LMWHs have different chemical structures, pharmacokinetics and anticoagulant activity, and their clinical application should not be replaced at will [15].

In general, administration of LMWHs does not require routine detection of anti-factor Xa activity due to the relatively stable pharmacokinetics of LMWHs. However, in patients with possible pharmacokinetic changes, mainly including gravidas, obese patients and patients with a history or potential of VTE, routine testing of anti-Xa activity is recommended [16]. Although many clinical research studies have been conducted on the efficacy of LMWHs in the treatment of RSA, most of them focused on the prevention of venous thromboembolism (VTE) [17–19]. Few studies examined the effect of different LMWHs on anti-Xa levels of RSA patients. In this study, we evaluated and compared the anticoagulant activity of different LMWHs by testing the activity of anti-Xa peak levels, and compared the adverse reactions.

## Materials and methods

### Patients

A single-center observational research was conducted in patients with RSA between 20 and 40 years old who visited the outpatient clinic of Renji Hospital affiliated to Shanghai Jiaotong University, Shanghai, China, from January 2020 to May 2021, with the following inclusion criteria: 1) a history of at least two consecutive spontaneous abortions; 2) heparin treatment during the pregnancy; and 3) blood test taken during pregnancy to measure anti-factor Xa. Patients were excluded from the study if they: 1) were under 20 or over 40 years old; 2) had a history of less than two consecutive spontaneous abortions or had two consecutive abortions with different partners; 3) did not complete the blood testing; 4) had major surgery in recent 3 years; and 5) had low AT level before pregnancy. In addition, all the patients underwent nucleic acid testing to exclude the infection of COVID-19. Written informed consent was provided by each participant. The study was approved by the Institutional Review Board of Ren Ji Hospital.

### Study design

All the participants began to receive subcutaneous administration of prophylactic dose LMWHs with 4100 IU nadroparin (Fraxiparine®, 0.4 ml: 4100 AXaIU, Aspen Inc.), 4000 IU enoxaparin (Clexane®, 0.4 ml: 4000 AXaIU, Sanofi Inc.) or 5000 IU dalteparin (Fragmin®, 0.2 ml: 5000 AXaIU, Pfizer Inc.) daily before pregnancy or upon initial diagnosis of pregnancy. They were divided into three groups according to the use of LMWHs: the nadroparin group, the enoxaparin group and the dalteparin group. Furthermore, all of the patients received patient education and were followed up in the way of periodic phone calls to ensure that treatments were been given in proper way. All of the patients underwent at least 6-week exposure of LMWHs before the blood test. In addition, each patient received low dose aspirin (less than 100 mg per day) daily during pregnancy. Anti-Xa peak level was measured 4 h after LMWH administration, so was the plasma level of AT-III and D-dimer. In addition, local allergy at the site of injection was recorded. Liver function, renal function and blood routine examination were measured every 4 weeks after pregnancy to assess adverse reactions, along with a thromboelastography (TEG) test to assess the coagulation status. Baseline characteristics were collected from medical records of the patients, including age, BMI before pregnancy, gestational week, the number of spontaneous miscarriages, history of live birth, plasma D-dimer, AT level, liver function, renal function and platelet count before pregnancy.

### Laboratory examination

Anti-Xa assay (normal range: 0.2–0.5 IU/ml for prophylactic dose and 0.5–1.2 IU/ml for therapeutic dose) was conducted in our laboratory by using Liquid Anti-Xa (Diagnostica Stago Inc., Parsippany, NJ, USA). Quantitative determination of plasma D-dimer (normal range: 0–0.5 mg/L) was performed via an automated latex enhanced immunoassay using a hemosIL DD kit (Instrumentation Laboratory Company, Bedford, MA, USA). AT-III (normal range: 75–125%) was measured by immunoturbidimetric method (Merck Inc., Darmstadt, HE, Germany). All tests were carried out according to the manufacturers’ instructions.

### Assessment on coagulation status

A TEG analyzer (TEG®5000, Haemoscope Inc., Boston, MA, USA) was used to assess the coagulation status. The reaction time (R), clot formation time (K), angle degree (α), maximum amplitude (MA) and coagulation index (CI) were recorded to evaluate the coagulation status of the patients. R represents the incubation period from the time when blood sample was placed on the TEG analyzer to initial fibrin formation, and it reflects the comprehensive effects of reactive coagulation factors. K and α represent the interaction between fibrin and platelet at the beginning of clot forming, and both reflect fibrinogen function. MA represents the maximum clot strength, which can reflect the function of platelet aggregation. CI is a combination of the indices, and reflects a comprehensive coagulation state of the reaction sample under various conditions.

### Adverse reactions

Common adverse reactions of LMWHs include local allergy, thrombocytopenia, liver lesion, renal dysfunction and osteoporosis. Local allergy includes ecchymosis, rash, pruritus, red and swollen and induration. Aspartate aminotransferase (ALT), alanine aminotransferase (AST) and total bile acids (TBA) were measured to assess patients’ liver function. An ALT > 40 U/L or AST > 40 U/L or TBA >  10 μmol/L was considered as liver lesion. Creatinine (Cr) and urea were tested to assess renal function. A Cr > 78 μmol/L or urea > 7.5 mmol/L was considered as renal dysfunction A routine blood test was conducted to monitor patients’ platelet count during pregnancy. Because of the particularity of gravidas, it is difficult to detect bone mineral density. Therefore, we only evaluated the other four adverse reactions.

### Statistical analysis

Statistical analyses were conducted by SPSS version 23.0 (IBM Corp., Armonk, NY, USA). The data were presented as the means ± SD or number (percentage). Comparisons among groups were made by Kruskal-Wallis H test or Chi-square test. In the presence of group difference, post-hoc Nemenyi test was used for pairwise comparison. Figures were generated by Prism version 9.0.0 (GraphPad Software, San Diego, CA, USA). *P* <  0.05 was considered statistically significant for all analyses.

## Results

### Baseline characteristics of the patients

**Fig. 1:**
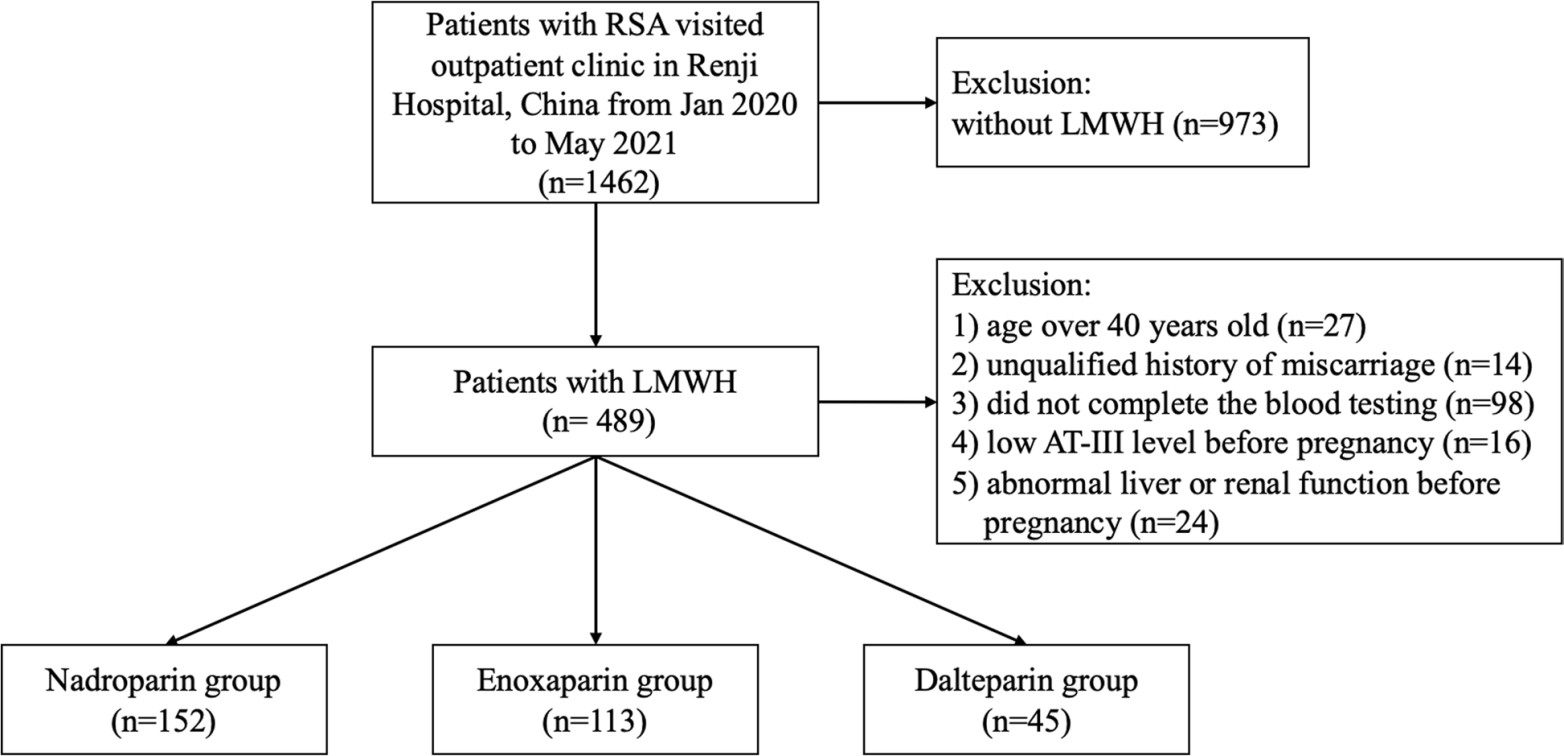
Flow chart for selection of study participants

A total of 1462 patients with RSA visited the outpatient department of Renji Hospital during January 2020 to May 2021. Among them, 310 patients satisfied the eligibility criteria and were includedin this study. These patients were divided into three groups according to the LMWHs they received: the nadroparin group (*n* = 152), the enoxaparin group (*n* = 113) and the dalteparin group (*n* = 45; Fig. 1). There were no significant differences among the three groups regarding age, BMI, previous miscarriage, previous live birth and gestational age. The liver and renal function and the platelet count of all the patients were within the normal range before pregnancy. Detailed information was presented in Table 1.

**Table 1:** Baseline characteristics of the study participants

Variable	Nadroparin group (***n*** = 152)	Enoxaparin group (***n*** = 113)	Dalteparin group (***n*** = 45)	***P*** value
Age (years, mean ± SD)	32 ± 3.4	32 ± 3.5	31 ± 3.5	0.20
≤ 30 years (n, %)	51 (33.6%)	36 (31.9%)	18 (40.0%)	0.62
30–35 years (n, %)	62 (40.7%)	41 (36.2%)	19 (42.2%)	0.69
≥ 35 years (n, %)	39 (25.7%)	36 (31.9%)	8 (17.8%)	0.18
BMI (kg/m^2^, mean ± SD)	22.2 ± 2.7	21.7 ± 2.7	21.8 ± 2.9	0.31
BMI > 24 kg/m^2^ (n, %)	32 (21.1%)	23 (20.4%)	9 (20.0%)	0.98
Previous miscarriages (median, IQR)	2 (2–6)	2 (2–6)	3 (2–4)	0.83
Previous live birth (n, %)	10 (6.6%)	9 (8.0%)	3 (6.7%)	0.90
Gestational age (days, mean ± SD)	117 ± 37.7	122 ± 39.8	111 ± 29.9	0.23

Data were presented as median (IQR), n (%) or mean ± SD

*BMI* body-mass index, *IQR* interquartile range, *SD* standard deviation

### Anti-Xa levels

**Fig. 2:**
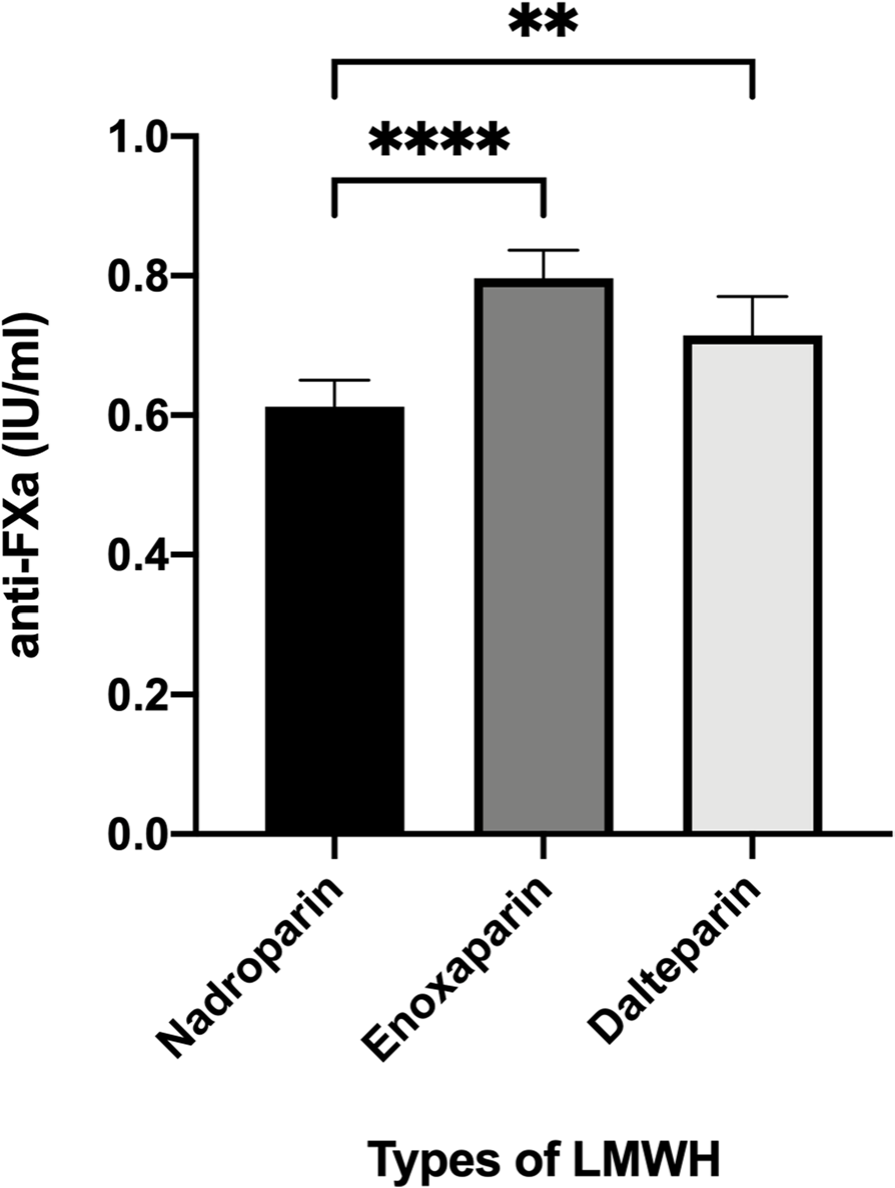
Mean anti-Xa levels at 4 h after LMWHs administration during pregnancy in patients with RSA. **P < 0.01, ****P < 0.0001

The anti-Xa level was significantly higher in the enoxaparin group (0.80 ± 0.22 IU/ml) and dalteparin group (0.71 ± 0.19 IU/ml), compared with the nadroparin group (0.61 ± 0.24 IU/ml; *P* < 0.0001 and *P* = 0.008, respectively), while the difference between enoxaparin group and dalteparin group was not statistically significant (*P* = 0.25; Fig. 2). We then analyzed the distribution of anti-Xa levels in each group. As presented in Table 2, within each individual group, most patients reached a therapeutic concentration of 0.5–1.2 IU/ml (105/152 in nadroparin group, 104/113 in enoxaparin group and 38/45 in dalteparin group, respectively), and almost all the remaining patients reached a prophylactic concentration of 0.2–0.5 IU/ml (44/47 in nadroparin group, 9/9 in enoxaparin group and 7/7 in dalteparin group, respectively).

**Table 2:** Distribution of anti-Xa levels

Anti-Xa levels	Nadroparin group (***n*** = 152)	Enoxaparin group (***n*** = 113)	Dalteparin group (***n*** = 45)
< 0.2 IU/ml	3 (2.0%)	0 (0%)	0 (0%)
0.2–0.5 IU/ml	44 (28.9%)	9 (8.0%)	7 (15.5%)
0.5–1.2 IU/ml	101 (66.5%)	101 (89.3%)	38 (77.8%)
> 1.2 IU/ml	4 (2.6%)	3 (2.7%)	0 (0%)

Results were presented as n (%)

**Fig. 3:**
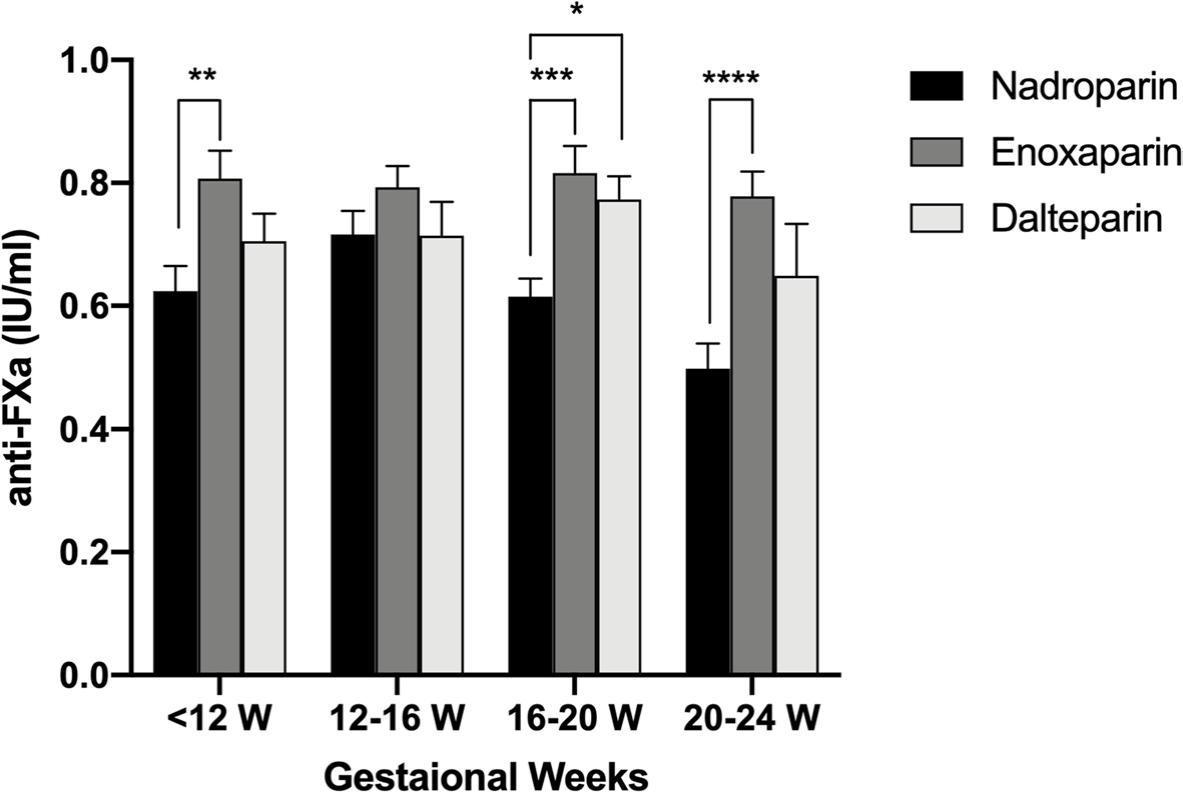
Subgroup (< 12 W, 12-16 W, 16-20 W, 20-24 W) analysis of mean anti-Xa levels at 4 h after LMWHs administration in patients with RSA. *P < 0.05, **P < 0.01, ***P < 0.001, ****P < 0.0001

A subgroup analysis was conducted according to the gestation age of the patients (< 12 W, 12–16 W, 16–20 W, and 20-24 W). The anti-Xa level was significantly higher in the enoxaparin group compared with the nadroparin group in patients with a gestation age < 12 W, 16–20 W and 20–24 W (*P* = 0.006, *P* = 0.0003 and *P* < 0.0001, respectively). The anti-Xa level in dalteparin group was significantly higher than the nadroparin group in patients with a gestation age of 16–20 W (*P* = 0.03). However, there was no significant difference between the enoxaparin group and the dalteparin group in each subgroup (Table 3 and Fig. 3). In addition, we found that the anti-Xa levels in the nadroparin group declined along with the pregnancy weeks: the anti-Xa level in 20–24 W was lower than < 12 W and 12–16 W (*P* = 0.01 and *P* < 0.0001, respectively). But there was no significant difference between the enoxaparin and dlateparin group in each subgroup.

**Table 3:** Subgroup analysis of anti-Xa levels according to gestational weeks

Gestational weeks	Nadroparin group	Enoxaparin group	Dalteparin group	***P*** value
< 12 W	0.62 ± 0.25 (*n* = 38)	0.81 ± 0.21 (*n* = 21)	0.71 ± 0.14 (*n* = 10)	0.006^a^
12–16 W	0.72 ± 0.22 (*n* = 34)	0.79 ± 0.19 (*n* = 31)	0.71 ± 0.21 (*n* = 14)	0.21
16–20 W	0.62 ± 0.19 (*n* = 44)	0.82 ± 0.23 (*n* = 27)	0.77 ± 0.13 (*n* = 12)	0.0003^a,^; 0.03^b^
20–24 W	0.50 ± 0.26 (*n* = 36)	0.78 ± 0.24 (n = 34)	0.65 ± 0.25 (*n* = 9)	< 0.0001^a^

Results were presented as mean ± SD; *SD* standard deviation

^a^ Nadroparin group vs. enoxaparin group

^b^ Nadroparin group vs. dalteparin group

### Assessment on coagulation status

**Fig. 4:**
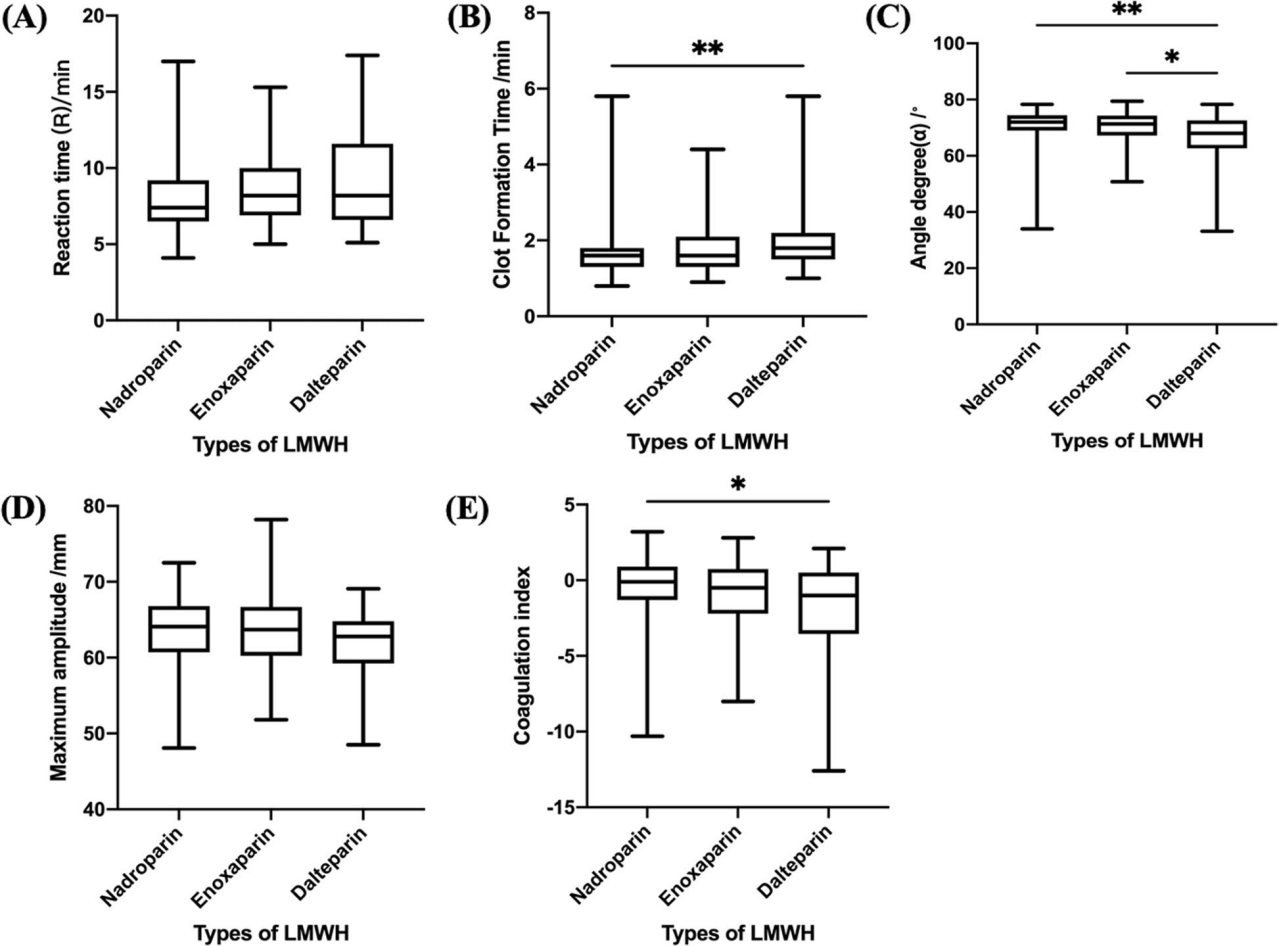
Comparison of thromboelastography (TEG) results among three different LMWHs in in patients with RSA. **A**, reaction time; **B**, clot formation time; **C**, angle degree; **D**, maximum amplitude; **E**, coagulation index. *P < 0.05, **P < 0.01

Compared with patients in the nadroparin group, those in the dalteparin group had significantly longer clot formation time (*P* = 0.003) and lower coagulation index (*P* = 0.02). Patients in the dalteparin group also had a significantly lower angle degree than the other two groups (*P* = 0.002 and *P* = 0.04 for comparison with the nadroparin group and the enoxaparin group, respectively; Fig. 4). Improved hypercoagulation in the dalteparin group, especially a decrease in fibrinogen levels, suggested that dalteparin exhibited a better anticoagulant effect (Table 4**)**.

**Table 4:** Impact of LMWHs on coagulation status

TEG Parameters	Nadroparin group (***n*** = 152)	Enoxaparin group (***n*** = 113)	Dalteparin group (***n*** = 45)	***P*** value
R time	8.09 ± 2.28	8.60 ± 2.42	9.40 ± 3.48	0.06
K time	1.64 ± 0.53	1.76 ± 0.58	2.08 ± 0.95	0.003^b^
Angle α	71.17 ± 5.03	70.26 ± 5.52	66.84 ± 8.53	0.002^b^; 0.04^,c^
MA	63.53 ± 4.52	63.25 ± 4.77	61.83 ± 4.71	0.11
CI	−0.51 ± 2.09	−0.98 ± 2.33	−2.06 ± 3.38	0.02^b^

Results were presented as mean ± SD

Normal range of R time, K time, angle α, MA and CI is 5–10 min, 1–3 min, 53–72°, 50–70 mm and − 3-3, respectively

*R time* reaction time, *K time* clot formation time, *Angle α* angle degree, *MA* maximum amplitude, *CI* coagulation index, *SD* standard deviation

^b^ Nadroparin group vs. dalteparin group

^c^ Enoxaparin group vs. dalteparin group

### Adverse reactions

The overall incidence rate of local allergy was 60.5% (92/152), 42.5% (48/113) and 33.3% (15/45) in the nadroparin group, enoxaparin group and daltaparin group, respectively. All of the allergy events were mild skin reactions, with no severe allergies. We found that enoxaparin and dalteparin exhibited a better performance regarding local allergy than the nadroparin group (Table 5). Examination by allergy type indicated that patients in the nadroparin group had a higher rate of pruritus than the enoxaparin group (30.9% vs. 9.7%; *P* < 0.0001) and the dalteparin group (30.9% vs. 6.6%; *P* < 0.0001), a higher rate of red and swollen (13.8% vs. 1.8%, *P* < 0.0001, and 13.8% vs. 2.2%, *P* = 0.03, respectivtly) and induration (42.1% vs. 20.3%, *P* < 0.0001, and 42.1% vs. 13.3%, *P* < 0.001, respectivtly). We found no significant difference among the three groups in ecchymosis and rash (*P* = 0.06 and *P* = 0.08, respectively; Table 5).

**Table 5:** Incidence of local allergy in each group

Local adverse reactions	Nadroparin group (***n*** = 152)	Enoxaparin group (***n*** = 113)	Dalteparin group (***n*** = 45)	***P*** value
Total	92 (60.5%)	48 (42.5%)	15 (33.3%)	0.004^a^; 0.002^b^
Ecchymosis	28 (18.4%)	34 (30.1%)	8 (17.8%)	0.06
Rash	7 (4.6%)	0 (0%)	2 (4.4%)	0.08^*^
Pruritus	47 (30.9%)	11 (9.7%)	3 (6.6%)	< 0.0001^a,b^
Red and swollen	21 (13.8%)	2 (1.8%)	1 (2.2%)	< 0.0001^a^; 0.03^b,*^
Induration	64 (42.1%)	23 (20.3%)	6 (13.3%)	< 0.0001^a^; < 0.001^b^

Results were presented as n (%)

*Fisher’s exact test

^a^ Nadroparin group vs. enoxaparin group

^b^ Nadroparin group vs. dalteparin group

The overall incidence of liver lesion was 10.5, 8 and 6.7% in the nadroparin group, enoxaparin group and dalteparin group, respectively, with no significant difference (*P* = 0.68; Table 6). All the patients with liver lesion only showed mild elevation of liver enzymes. No severe hepatic insufficiency was observed. Renal dysfunction was rare.

**Table 6:** Incidence of liver and renal lesion and thrombocytopenia in each group

Adverse reactions	Nadroparin group (***n*** = 152)	Enoxaparin group (***n*** = 113)	Dalteparin group (***n*** = 45)	***P*** value
Liver lesion (n, %)	16 (10.5%)	9 (8.0%)	3 (6.7%)	0.68^*^
Renal lesion (n, %)	1 (0.66%)	1 (0.88%)	0 (0%)	NA
HIT (n, %)	0 (0%)	0 (0%)	0 (0%)	NA
PLT count decrease > 30%	15 (9.9%)	7 (6.2%)	5 (11.1%)	0.48

Results were presented as n (%) or mean ± SD

*HIT* heparin induced thrombocytopenia, *PLT* platelet, *NA* not available

* Fisher’s exact test

There was no patient with heparin-induced thrombocytopenia (HIT). The proportion of > 30% decrease in platelet count from the baseline was 9.9% (15/152), 6.2% (7/113) and 11.1% (5/45) in the nadroparin group, enoxaparin group and dalteparin group, respectively, with no significant difference among the three groups (*P* = 0.48; Table 6).

## Discussion

In this study, we compared the effects of three most commonly used LMWHs on anti-Xa levels, coagulation status and adverse reaction. We found that patients in the enoxaparin group exhibited higher and more stable anti-Xa levels along gestation than the nadroparin group, although nearly all of the patients reached a prophylactic concentration. Furthermore, patients in the nadroparin group had higher rate of local allergy compared with the enoxaparin group and the dalteparin group. All the patients had low incidence rate of liver and renal dysfunction, and thrombocytopenia. To the best of our knowledge, this is the first study to compare the three LMWHs regarding anti-Xa levels and adverse reactions. Our results provide important information for the selection of optimal LMWH to treat RSA.

The different LMWHs used in our study were derived from standard commercial grade UFH by enzymatic or chemical depolymerization. The production of each LMWH used a unique proprietary manufacturing process resulting in distinct structural features. For example, nitrous acid depolymerization was used in the preparation of nadroparin and dalterparin characteristerized by the presence of 2,5-anhydro-D-mannose at reducing terminus [15]. By constrast, benzylation followed by alkaline depolymerization was used in the manufacture of enoxaparin characterized by the presence of 4,5 unsaturated uronic acid at non-reducing terminus [20]. These subtle differences in chemical structure might have an influence on the physicochemical properties of the LMWHs, which might account for the observed difference in the performance of the three LMWHs [21]. More studies are needed to further explore the exact physiological mechanisms underlying varied effect of the LMWHs on anti-Xa levels.

Most previous research studies evaluating the relationship between LMWHs and anti-Xa levels in pregnancy focused on pharmacokinetics of LMWH or LMWH dose adjustment [22–24]. The anti-Xa levels of prophylactic dose (1 dose per day) in existing studies mostly fell in 0.2–0.5 IU/ml [23–26], while in our study, most cases reached a therapeutic concentration of 0.5–1.2 IU/ml. The inconsistent findings regarding anti-Xa levels might be due to the difference in patient characteristics. Specifically, the mean weight and BMI of the patients in our study was only 57.0 kg and 21.9 kg/m^2^, respectively. By contrast, previous studies on patients in western countries were relatively more obese, with a mean weight ranging from 67 to 85.3 kg, and a mean BMI ranging from 25.7 to 28.8 kg/m^2^ [27, 28]. As a result, the LMWH dosage per unit weight of our patients was larger than that of previous studies. In addition, LMWHs were mainly used to prevent VTE events in previous studies, as most of the included patients had a history of VTE or had potential risk of VTE [18, 26]. In our study, LMWHs were used to treat RSA and none of our patients had a history of VTE. Together, these difference might partly account for the difference in anti-Xa levels.

Previous studies suggested that dosage of LMWH should be adjusted according to the anti-Xa levels [26] and should be increased along gestation [29]. Another retrospective research also found that fixed-dosage regimen of enoxaparin could not reach the target anti-Xa levels in gravidas [27]. By contrast, in our study, although the anti-Xa levels in the nadroparin group declined with the progress of pregnancy, almost all the patients reached a target concentration. The difference in findings might be due to difference in the ethnicity of study participants. Moreover, different from previous studies, our study excluded patients who had low AT-III levels at baseline. LMWHs can only exert their anticoagulant activity by binding to AT-III to reach a marked effect [12]. LMWH resistance can be minimized by excluding patients with low AT-III levels, leading to a higher mean anti-Xa levels as observed in our study.

Local allergy caused by LMWHs is a delayed-type, non-IgE-mediated hypersensitivity response and should be taken seriously [30]. An earlier systematic review revealed that the incidence of local allergy in LMWH ranged from 7.5 to 39%, with a higher rate in gravidas [31]. Moreover, a previous RCT research showed that almost half of the women who received nadroparin therapy had skin allergy, with ecchymosis, pruritus and swollen being the most common [32]. Furthermore, a prospective research found that patients who used nadroparin had a higher rate of local allergy than those who used enoxaparin and dalteparin [28]. Consistent with these finding, we also observed a high rate of skin allergy (50% in total), especially in the nadroparin group (60.5%). Together, these observations highlight that nadroparin might lead to a high risk of skin allegry.

Our study has some limitations. The sample size of each group was relatively small, especially the dalteparin group, which may bias the results. All the patients received self subcutaneous injection of LMWHs, although they received relevant training before the study. This may partly account for a relatively higher rate of local allergy and could possibly influence anti-Xa levels. Randomized controlled trials with larger sample sizes are needed to validate the findings of our study.

## Conclusion

In conclusion, we found that enoxaparin exhibited a better performance regarding anti-Xa levels in that patients who received enoxaparin had stable anti-Xa levels during pregnancy and had lower incidence of adverse reaction. In addition, a prophylactic dose of LMWH is sufficient and safe in the treatment of RSA in Chinese patients. More propsective research studies are needed to validate our results and explore generalizability of the findings to patients of different ethnicities.

## Data Availability

The dataset generated and analyzed during the current study is not publicly available as it was derived from patient medical records, but the data are available from the corresponding author on reasonable request.

## Abbreviations

RSA: Recurrent spontaneous abortion
RM: Recurrent miscarriages
LMWH: Low molecular weight heparin
PTS: Prethrombotic state
UFH: Unfractionated heparin
VTE: Venous thromboembolism
TEG: Thromboelastography

## References

[1] Bhattacharya S, Townend J, Shetty A, Campbell D, Bhattacharya S (2008). Does miscarriage in an initial pregnancy lead to adverse obstetric and perinatal outcomes in the next continuing pregnancy?. BJOG.

[2] Rai R, Regan L (2006). Recurrent miscarriage. Lancet.

[3] Wu M, Liu P, Cheng L (2015). Galectin-1 reduction and changes in T regulatory cells may play crucial roles in patients with unexplained recurrent spontaneous abortion. Int J Clin Exp Pathol.

[4] Larsen EC, Christiansen OB, Kolte AM, Macklon N (2013). New insights into mechanisms behind miscarriage. BMC Med.

[5] Christiansen OB, Nybo Andersen AM, Bosch E, Daya S, Delves PJ, Hviid TV, Kutteh WH, Laird SM, Li TC, van der Ven K (2005). Evidence-based investigations and treatments of recurrent pregnancy loss. Fertil Steril.

[6] Pagnini I, Simonini G, Cavalli L, la Marca G, Iuliano A, Brandi ML, Bellisai F, Frediani B, Galeazzi M, Cantarini L (2014). Bone status of children born from mothers with autoimmune diseases treated during pregnancy with prednisone and/or low molecular weight heparin. Pediatr Rheumatol Online J.

[7] Shomer E, Katzenell S, Zipori Y, Rebibo-Sabbah A, Brenner B, Aharon A (2016). Microvesicles of pregnant women receiving low molecular weight heparin improve trophoblast function. Thromb Res.

[8] Luley L, Schumacher A, Mulla MJ, Franke D, Lottge M, Fill Malfertheiner S, Tchaikovski SN, Costa SD, Hoppe B, Abrahams VM (2015). Low molecular weight heparin modulates maternal immune response in pregnant women and mice with thrombophilia. Am J Reprod Immunol.

[9] Xu GL, Hu XF, Han YM, Wei AW (2018). Clinical efficacy of low molecular heparin on unexplained recurrent spontaneous abortion. Clin Lab.

[10] Rottenstreich A, Amsalem H, Kleinstern G, Kalish Y (2017). Outcomes of threatened abortions after anticoagulation treatment to prevent recurrent pregnancy loss. Reprod BioMed Online.

[11] Hirsh J, Anand SS, Halperin JL, Fuster V, American Heart A (2001). Guide to anticoagulant therapy: heparin : a statement for healthcare professionals from the American Heart Association. Circulation.

[12] Gray E, Mulloy B, Barrowcliffe TW (2008). Heparin and low-molecular-weight heparin. Thromb Haemost.

[13] Spadarella G, Di Minno A, Donati MB, Mormile M, Ventre I, Di Minno G (2020). From unfractionated heparin to pentasaccharide: paradigm of rigorous science growing in the understanding of the in vivo thrombin generation. Blood Rev.

[14] Onishi A, St Ange K, Dordick JS, Linhardt RJ (2016). Heparin and anticoagulation. Front Biosci (Landmark Ed).

[15] Hirsh J, Raschke R (2004). Heparin and low-molecular-weight heparin: the seventh ACCP conference on antithrombotic and thrombolytic therapy. Chest.

[16] Laposata M, Green D, Van Cott EM, Barrowcliffe TW, Goodnight SH, Sosolik RC (1998). College of American Pathologists Conference XXXI on laboratory monitoring of anticoagulant therapy: the clinical use and laboratory monitoring of low-molecular-weight heparin, danaparoid, hirudin and related compounds, and argatroban. Arch Pathol Lab Med.

[17] Dranitsaris G, Shane LG, Woodruff S (2019). Low-molecular-weight heparins for the prevention of recurrent venous thromboembolism in patients with cancer: a systematic literature review of efficacy and cost-effectiveness. J Oncol Pharm Pract.

[18] Droege ME, Mueller EW, Besl KM, Lemmink JA, Kramer EA, Athota KP, Droege CA, Ernst NE, Keegan SP, Lutomski DM (2014). Effect of a dalteparin prophylaxis protocol using anti-factor Xa concentrations on venous thromboembolism in high-risk trauma patients. J Trauma Acute Care Surg.

[19] Rathbun SW, Aston CE, Whitsett TL (2012). A randomized trial of dalteparin compared with ibuprofen for the treatment of superficial thrombophlebitis. J Thromb Haemost.

[20] Hao C, Sun M, Wang H, Zhang L, Wang W (2019). Low molecular weight heparins and their clinical applications. Prog Mol Biol Transl Sci.

[21] Ingle RG, Agarwal AS (2014). A world of low molecular weight heparins (LMWHs) enoxaparin as a promising moiety--a review. Carbohydr Polym.

[22] Friedrich E, Hameed AB (2010). Fluctuations in anti-factor Xa levels with therapeutic enoxaparin anticoagulation in pregnancy. J Perinatol.

[23] Boban A, Paulus S, Lambert C, Hermans C (2017). The value and impact of anti-Xa activity monitoring for prophylactic dose adjustment of low-molecular-weight heparin during pregnancy: a retrospective study. Blood Coagul Fibrinolysis.

[24] Fox NS, Laughon SK, Bender SD, Saltzman DH, Rebarber A (2008). Anti-factor Xa plasma levels in pregnant women receiving low molecular weight heparin thromboprophylaxis. Obstet Gynecol.

[25] Rowan JA, McLintock C, Taylor RS, North RA (2003). Prophylactic and therapeutic enoxaparin during pregnancy: indications, outcomes and monitoring. Aust N Z J Obstet Gynaecol.

[26] Shapiro NL, Kominiarek MA, Nutescu EA, Chevalier AB, Hibbard JU (2011). Dosing and monitoring of low-molecular-weight heparin in high-risk pregnancy: single-center experience. Pharmacotherapy.

[27] Stock SJ, Walker MC, Edelshain BT, Horn L, Norman JE, Denison FC (2011). Fixed dosing regimes of enoxaparin for thromboprophylaxis do not reliably achieve target anti-Xa levels in women of normal weight during pregnancy. Eur J Obstet Gynecol Reprod Biol.

[28] Schindewolf M, Gobst C, Kroll H, Recke A, Louwen F, Wolter M, Kaufmann R, Boehncke WH, Lindhoff-Last E, Ludwig RJ (2013). High incidence of heparin-induced allergic delayed-type hypersensitivity reactions in pregnancy. J Allergy Clin Immunol.

[29] Quinn J, Von Klemperer K, Brooks R, Peebles D, Walker F, Cohen H (2009). Use of high intensity adjusted dose low molecular weight heparin in women with mechanical heart valves during pregnancy: a single-center experience. Haematologica.

[30] Schindewolf M, Lindhoff-Last E, Ludwig RJ, Boehncke WH (2012). Heparin-induced skin lesions. Lancet.

[31] Schindewolf M, Recke A, Zillikens D, Lindhoff-Last E, Ludwig RJ (2017). Nadroparin carries a potentially high risk of inducing cutaneous delayed-type hypersensitivity responses. Contact Dermatitis.

[32] Kaandorp SP, Goddijn M, van der Post JA, Hutten BA, Verhoeve HR, Hamulyak K, Mol BW, Folkeringa N, Nahuis M, Papatsonis DN (2010). Aspirin plus heparin or aspirin alone in women with recurrent miscarriage. N Engl J Med.

